# Trypanosomiasis in the Congo

**Published:** 1904-10-15

**Authors:** 


### TRYPANOSOMIASIS IN THE CONGO.

The Liverpool School of Tropical Medicine has recently issued a valuable series of reports on the trypanosomes of the Congo State, and on the symptoms and course of the disease occasioned by them. The discovery of trypanosomes in the blood and cerebro-spinal fluid of the sufferers from sleeping-sickness in Uganda, and the prevalence of what appeared to be the same malady in the Congo State, induced the King of the Belgians to invite the authorities of the Liverpool School to investigate the question; and Drs. Dutton, Todd, and Christy were appointed a Commission for the purpose. The general result of their inquiries has been to show that veritable "sleeping-sickness" is less common in the Congo than had been assumed; many of the cases presented to the Commissioners being of other forms of chronic disease; but a sufficient number of instances of actual trypanosomiasis were observed, and it was found that the somnolence from which the malady has obtained its trivial name is by no means a universal symptom among the persons suffering or even dying from it. Trypanosomes were often discovered in the peripheral blood taken from finger punctures, but their presence was curiously uncertain, and they would be absent for days together without any manifest reason for either their absence or their return. They were discovered also in the cerebro-spinal fluid obtained by fine puncture in the lumbar region, and the way in which these punctures were made and the precautions by which the fluid was obtained without admixture of blood, are fully described in the report. Perhaps the most interesting of the discoveries announced by the Commission is that the conveyance of the trypanosome parasites from person to person in the Congo State may possibly not be ordinarily effected by complete flies, as in the case of the kindred equine disease disseminated by the tsetse, but by the blood-sucking larva of a fly which is extensively distributed in tropical and sub-tropical Africa, and which has been identified by Mr. Austen, of the British Museum, as

*Auchmeromyia luteola.* These larvæ are active during the night, and hide during the day in the cracks of the floors of the native huts, whence they may be dug out full of the blood which they have taken from sleepers in the room. Many experiments have been made by the Commission in the hope of discovering some medicinal agent by which trypanosomes may be killed in the human circulation, as the ague parasites are killed by quinine, but these, so far, have been only partially successful. The best results have been obtained from combinations of arsenic with an aniline dye named trypan red; but the whole therapeutical question demands more extended investigation.

